# Tuning of defects in ZnO nanorod arrays used in bulk heterojunction solar cells

**DOI:** 10.1186/1556-276X-7-655

**Published:** 2012-11-27

**Authors:** Diana C Iza, David Muñoz-Rojas, Quanxi Jia, Brian Swartzentruber, Judith L MacManus-Driscoll

**Affiliations:** 1 Department of Materials Science and Metallurgy, University of Cambridge, Pembroke Street, Cambridge, CB2 3QZ, UK; 2 MPA CINT, MS K771, Los Alamos National Laboratory, Los Alamos, NM 87545, USA; 3 Sandia National Laboratories, MS 1303.1515 Eubank SE, Albuquerque, NM 87123, USA

**Keywords:** ZnO, Solar cells, Bulk heterojunction, Photoluminescence, Defects

## Abstract

With particular focus on bulk heterojunction solar cells incorporating ZnO nanorods, we study how different annealing environments (air or Zn environment) and temperatures impact on the photoluminescence response. Our work gives new insight into the complex defect landscape in ZnO, and it also shows how the different defect types can be manipulated. We have determined the emission wavelengths for the two main defects which make up the visible band, the oxygen vacancy emission wavelength at approximately 530 nm and the zinc vacancy emission wavelength at approximately 630 nm. The precise nature of the defect landscape in the bulk of the nanorods is found to be unimportant to photovoltaic cell performance although the surface structure is more critical. Annealing of the nanorods is optimum at 300°C as this is a sufficiently high temperature to decompose Zn(OH)_2_ formed at the surface of the nanorods during electrodeposition and sufficiently low to prevent ITO degradation.

## Background

Bulk heterojunction (BHJ) solar cells based on composites of electron-donating and electron-accepting organic semiconductors have high performances, reaching efficiencies of over 8% [1-3]. The incorporation of metal oxides as blocking layers improves device performance by imposing charge selectivity at the collecting electrode as well as by preventing shorting in the devices [4-7]. Other benefits include improving stability of the polymers due to the absorption of UV light [8]. ZnO has attracted particular interest for hole blocking layers due to its intrinsic high carrier mobility and ease of processing in nanostructured form [9-13].

Low-cost manufacturing routes for metal oxides, such as electrochemical deposition and hydrothermal processes, offer realistic scale-up possibilities for low-cost photovoltaics [14]. In the case of electrodeposition, the advantages of the simplicity of using aqueous solutions and the low energy requirement of the deposition method are balanced against achievement of only moderate crystalline quality. Indeed, post-deposition heat treatments are necessary to reduce defect concentrations and to prevent high series resistance in the final photovoltaic device. It is well known that the defect chemistry of ZnO is complex, with a range of oxygen or zinc defects of varying charges and with concentrations that are interdependent on each other [15,16]. With particular relevance to their use in solar cells, it is important to understand the influence of the defects on photovoltaic performance. However, despite the wide impact of use of metal oxides in organic solar cells [17-19], surprisingly few studies of this nature have so far been undertaken [20-22]. Herein, we present a detailed study of how different annealing environments influence the defect types in ZnO nanorods and also how they influence the performance of bulk heterojunction solar cells.

## Methods

### ZnO nanorod growth and annealing

Large-scale ZnO nanorod arrays on ITO glass substrates were grown by a simple one-step electrodeposition method. A 1.4 × 1.4 cm^2^ ITO/glass substrate (Praezisions Glas & Optik, GmbH, Iserlahn, Germany; 180 nm ITO on float glass and sheet resistance of *ca*. 10 Ω/sq.) was used as the working electrode and a 4-cm^2^ Pt foil as the counter electrode. A 0.01 M Zn(NO_3_)_2_ solution was used. Growth was carried out galvanostatically at a constant current density of 0.15 mA cm^−2^ at 85°C.

The morphology of the electrodeposited ZnO depends on the solution concentration used [23-25]. In order to obtain nanorods without the need of a seed layer, a solution of intermediate concentration (0.01 M Zn(NO_3_)_2_) was used, thus simplifying the device fabrication.The arrays obtained with this concentration are sufficiently dense to ensure that short circuiting in the solar cell devices is prevented. During electrodepositon, a Zn(OH)_2_ film seed layer is initially obtained. Once this layer is formed, nanorods form by decomposition of this Zn(OH)_2_ with nucleation believed to occur after hydroxide dehydration [26]. At the end of electrodeposition, a ZnO nanorod array is obtained, with Zn(OH)_2_ being present at the surface of the nanorods.

Post-annealing studies were performed on the as-deposited nanorod arrays at either 100°C, 200°C, 300°C, 400°C, or 500°C for 4 h with heating and cooling rates of 1°C min^−1^ and 3°C min^−1^, respectively.

The annealing atmosphere was either pure air or air saturated with Zn vapor (formed by wrapping Zn foil around the samples). Annealing in these different atmospheres was performed with the aim of differentiating between the influences of oxygen vacancies and Zn-related defects [27,28] on BHJ cell performance. It is well known that Zn annealing can change either the Zn interstitial and/or Zn vacancy concentration, depending on the form of the starting ZnO material and hence the initial defect landscape and also on the presence of H and N impurities [16,29].

### ZnO characterization

To assess defect types and concentrations, *PL measurements* were performed at room temperature with an ACCENT RPM 2000 compound semiconductor PL system equipped with a Nd:YAG laser of wavelength 266 nm. The area under the visible band emission was calculated in order to estimate changes in the defect concentration.

*IR measurements* were undertaken to determine the information about the decomposition of Zn(OH)_2_ present on our nanorod samples. A Bruker 66v IFS spectrometer (Brookline, MA, USA) was used with a KBr beamsplitter, a Globar source, and a DTGS detector. The arrays were grown on quartz substrates onto which ITO was sputtered using a K575 Emitech sputter coater (Ashford, UK), and the samples were analyzed under vacuum. The data were recorded with an instrumental resolution of 2 cm^−1^ and 512 scans.

*Electrical measurements* of ZnO nanorods (on ITO on glass) were performed using a two-probe nanomanipulator retrofit inside a JEOL 6701F scanning electron microscope (Akishima, Tokyo, Japan). Current versus voltage curves were acquired by making a contact to the top of a ZnO nanorod with one of the probes, applying a bias between the probe and the substrate and measuring the current flowing through the rod. The current and voltage to the probes and the sample were independently measured and controlled using an Agilent B1500A semiconductor device analyser (Santa Clara, CA, USA). The resistances were determined for several rods at each temperature and the values averaged. For the calculation of the resistivities, a rod length of 800 nm was estimated from SEM images (the deviation from the average being around 5%).

*Scanning electron microscopy* images were taken using a LEO VP-1530 field emission scanning electron microscope (Peabody, MA, USA).

### Photovoltaic cell processing

ZnO nanorod arrays were incorporated in inverted poly(3-hexylthiophene):phenyl-C61-butyric acid methyl ester (P3HT:PCBM) bulk heterojunction cells. Prior to spin coating of the thin blend, the arrays were annealed in air in a tubular furnace as described above.

### Solar cell measurements

Current density-voltage measurements of all devices were performed using a Keithley 2636 source meter (Cleveland, OH, USA) with a custom-made Lab-View program. A Newport Oriel class A solar simulator (Irvine, CA, USA) equipped with AM 1.5 G filters calibrated to a silicon reference diode was used at 100 mW cm^−2^ intensity. Several cells were studied.

**Figure 1:**
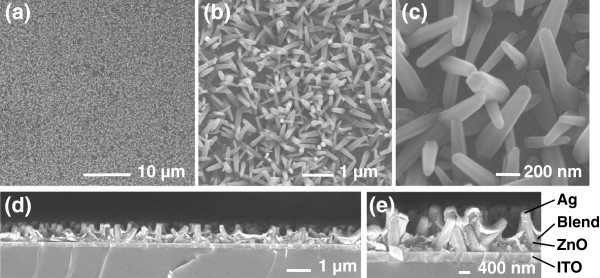
**SEM and cross-sectional images.** (**a**) to (**c**) SEM images of ZnO nanorod arrays deposited on bare ITO. (**d**) to (**e**) Cross-sectional images of ITO/ZnO/P3HT:PCBM/Ag devices.

Figure 1a,b,c shows the scanning electron micrograph (SEM) images of the ZnO nanorods produced. Uniform coverage of the ITO/glass substrate with the nanorod arrays was obtained. The nanorods are 80 to 130 nm in diameter and *ca.* 800 nm in length. Figure 1d,e shows cross-sectional images of the solar cell devices produced herein, which will be discussed later.

## Results and discussion

**Figure 2:**
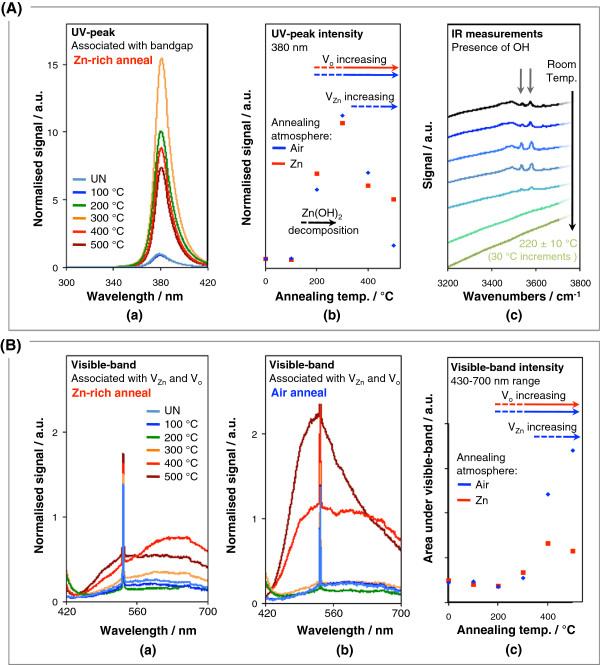
**PL data.** Panel (**A**): (**a**) PL UV-peak of nanorods annealed at different temperatures in a Zn-rich atmosphere, (**b**) UV-peak intensity as a function of annealing temperature for samples annealed in a Zn-rich atmosphere and air, (**c**) Infrared spectroscopy of ZnO nanorod arrays as a function of temperature. Panel (**B**): (**a**) and (**b**) PL visible band of nanorods annealed at different temperatures in a Zn-rich atmosphere and air, respectively, and (**c**) area under PL visible band as a function of annealing temperature for samples annealed in a Zn-rich atmosphere and air. Defect evolution trends are indicated by a red arrow for annealing in Zn atmosphere and by a blue arrow for annealing in air. UN indicates un-annealed.

**Figure 3:**
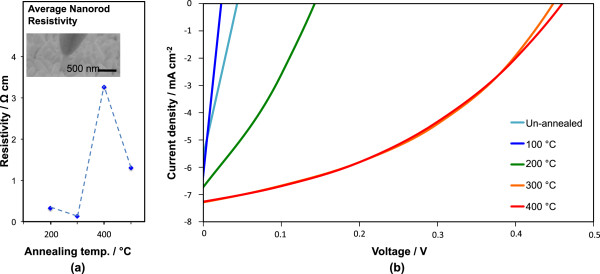
**Resistivity of the nanorods and *J*-*V *responses of the devices.** (**a**) Resistivity versus temperature plot of nanorods annealed in air. **(b)***J*-*V* responses of the devices as a function of annealing temperature of the ZnO. Annealing was undertaken in air.

Firstly, we present the PL data on our samples together with IR measurements (Figure 2). We then study the resistivity of the nanorods (Figure 3a) and the photovoltaic performance of BHJ cells incorporating the differently annealed nanorods (Figure 3b) in relation to the findings of Figure 2.

A typical PL spectra of ZnO consists of at least the bandgap UV peak and a visible band emission [26,30-36]. To aid clarity, we consider the PL data in two wavelength regions. The UV peak (300 to 420 nm) is shown in panel A and the visible band (420 to 700 nm) in panel B.

### Photoluminescence UV peak

The UV peak is associated with free-exciton recombination across the bandgap, its intensity being higher when there are less recombination traps within the bandgap. Figure 2A (a and b) shows the evolution of the UV peak as a function of annealing temperature and atmosphere together with the evolution of peaks in the IR measurements (c).

The as-deposited nanorods have a low UV peak intensity (part a of Figure 2A). This has been attributed previously to signal quenching from a Zn(OH)_2_ layer present on the as-deposited ZnO [30,37,38]. Indeed, the presence of OH peaks in the IR spectra at *ca.* 3,550 and 3,580 cm^−1^[31,39,40] are very clear (part c of Figure 2A). After annealing the nanorods at 100°C, there was no significant change in the UV peak emission intensity with respect to the un-annealed sample. This is consistent with the annealing temperature being too low for the Zn(OH)_2_ to dehydrate to ZnO.

From 100°C to 200°C, the UV peak increases in intensity (parts a and b of Figure 2A). This is in agreement with the IR spectra (part c of Figure 2A) which shows the Zn(OH)_2_ gradually decomposing to ZnO + H_2_O until the peaks are no longer visible by approximately 160°C, the reported decomposition temperature of Zn(OH)_2_[41]. Note that the removal of water from the sample is observed clearly as a reduction in the background hump in part c of Figure 2A.

From 200°C to 300°C, the PL peak intensity continues to increase (part b of Figure 2A) which is expected as the crystallinity of the ZnO increases with temperature. However, from 300°C to 500°C, the intensity decreases. Clearly, the crystallinity of the ZnO will continue to improve at above 300°C, but point defects in the form of oxygen and zinc vacancies will also form as the annealing temperature is increased [27,30,33,38]. Several works have been undertaken to analyze the kinetics of point defects in ZnO as a function of temperature [15,42-45], with zinc vacancies being proposed to have the lowest formation energy in n-type ZnO [43].

To distinguish between zinc vacancies and oxygen vacancies, we study the difference in PL spectra between air-annealed and Zn-annealed samples. From 400°C to 500°C, there is a sharp decrease in PL UV peak intensity indicating a strong increase in defect density. At 500°C, the higher intensity of the PL UV peak for the Zn-annealed sample compared to the air-annealed sample (reproduced for several samples) indicates that there is filling of zinc vacancies. Since there is little difference between the data sets at 400°C, this confirms that the kinetics for Zn in-diffusion is rapid enough only above this temperature [27,28]. Whether, upon Zn ingress into ZnO, zinc vacancies are filled or Zn interstitial concentration is increased depends on the starting cation stoichoimetry of the sample [16]. We deduce that the electrodeposited samples here are Zn deficient to begin with, which concurs with the oxidizing nature of the growth environment [15].

### Photoluminescence visible band

Specific energies of emission within the visible band have been ascribed to a variety of defects in ZnO at different energies. However, there is no generally accepted consensus about specific defect-energy relations [16,30].

After annealing from the growth temperature to 200°C, for both air and Zn-annealing atmospheres, there is a decrease in the visible band integrated intensity (Figure 2B). This is a result of the Zn(OH)_2_ decomposition to ZnO, consistent with part b of Figure 2A and with previous findings [30,37,38]. On the other hand, from 200°C to 300°C, the intensity increases (part c of Figure 2B) indicating an increase in point defect concentration. From 300°C to 400°C, the intensity increases for both air- and zinc-annealed samples, but a considerably higher intensity results for the air-annealed sample indicating that Zn vacancies contribute to the band. At 500°C, the air-annealed sample intensity increases further, but the Zn-annealed sample intensity decreases. These trends indicate that for the air-annealed sample, *both* oxygen vacancy and Zn vacancy concentrations increase with temperature, but that for the Zn annealed sample, while the oxygen vacancy concentration increases, above 400°C the Zn vacancy concentration decreases, which is in agreement with the trend observed for the UV peak at 500°C where the air-annealed and Zn-annealed points diverge (part b of Figure 2A).

As mentioned previously, more than one defect is responsible for the visible band emission [30,33-36,46]. Indeed, this is why a band is observed rather than a single peak. The intensities of the bands for the air- and Zn-annealed samples (parts a and b of Figure 2B) were compared to determine which defect emission contributed to which wavelength region within the bands. Firstly, it is observed that at 400°C and above, the shapes and intensities of the bands differ more from one another compared to the lower temperature data. For the Zn-annealed sample (part a of Figure 2B), the longer emission wavelength (at approximately 630 nm) decreased in intensity on going from 400°C to 500°C, which indicates that the 630-nm emission arises from Zn vacancies. Hence, as the kinetics for Zn ingress into the sample is enhanced by increasing the temperature, more Zn vacancies are filled; thus, the defect concentration decreases and the peak intensity decreases. For the air-annealed sample, the intensity of the 630-nm emission does not show the same marked decrease in intensity from 400°C to 500°C (part b of Figure 2B) because excess Zn is not available in the annealing atmosphere to fill the vacancies. Ascribing the 630-nm emission to zinc vacancies is in agreement with [47] although other reports quote other energies for emissions resulting from zinc vacancies [30].

Elucidation of the origin of the 530-nm emission is made, again, by observing emission intensity differences for the different annealing atmospheres on going from 400°C to 500°C. We observe that the intensity increases steadily both for the Zn-annealed (part a of Figure 2B) and air-annealed (part b of Figure 2B) samples, indicating that this emission arises from oxygen vacancies which increase in concentration with temperature, in agreement with [29] and [48].

### Nanorod resistivities

The resistivities averaged for several nanorods on ITO after air annealing are shown in Figure 3a. The inset of Figure 3a shows an image of a micron-sized nanoprobe contacting to a single nanorod among the array of nanorods. The lowest resistivity among the samples is at 300°C which is coincident with the highest UV peak intensity. The trend is as expected since increasing the concentration of electron-donating oxygen vacancies and improving crystallinity with temperature leads to a reduction in ZnO resistivity [27,49]. The higher resistivities for the 400°C and 500°C samples concur with the sharply decreased UV peak intensity (parts a and b of Figure 2A) and increased defect concentrations causing more carrier scattering [50].

### Photovoltaic measurements on bulk heterojunction cells incorporating ZnO nanorods

Cross-sectional images of the hybrid cells are shown in Figure 1d,e, and the typical current density-voltage (*JV*) response under an AM 1.5 G solar simulator for cells annealed up to 400°C is shown in Figure 3b. Data for cells annealed at 500°C are not included since there was a wide spread in the data from one sample to another consistent with significant ITO degradation at this temperature [51-54]. Zn-annealed nanorods were not studied in the cells as they give different defect concentrations only at 400°C and above, and at these temperatures, the problems of ITO degradation mean that any benefits of lower zinc vacancy concentration are outweighed by increased device processing complexity and higher cell resistivity.

For the un-annealed and 100°C annealed samples, a linear *JV* curve was obtained showing little rectification. A considerably better response was observed for arrays annealed at 200°C and above when the Zn(OH)_2_ layer had fully decomposed (part c of Figure 2A). The presence of Zn(OH)_2_ prevents the formation of a clean interface between hydrophobic P3HT:PCBM and the ZnO, thus stopping efficient charge separation from taking place. The Zn(OH)_2_ is also likely to be acting as a recombination region and possible resistive barrier layer [26].

At above 200°C, the crystallinity of the ZnO increases sharply (parts a and b of Figure 2A) as the surface hydroxide layer has already vanished. Hence, the cell performance improves as expected. By 300°C (where the UV peak intensity is at a maximum and the average nanorod resistivity is at a minimum (Figure 3a)), the short-circuit current density (*J*_sc_) and open-circuit voltage (*V*_oc_) values peak (Figure 3b and Table 1).

**Table 1:** Photovoltaic cell performances obtained for ITO/ZnO/P3HT:PCBM/Ag devices containing ZnO nanorods annealed in air at different temperatures

**Annealing temperature of ZnO array (°C)**	**Open-circuit voltage (V)**	**Short-circuit current density (mA cm**^**−2**^**)**	**Fill factor (%)**	**Efficiency (%)**
As-deposited	0.04	5.60	24.76	0.06
100	0.02	6.25	24.53	0.04
200	0.14	6.72	28.20	0.27
300	0.45	7.28	40.96	1.34
400	0.46	7.26	39.42	1.32

The ZnO nanorod arrays were annealed to different temperatures before cell fabrication.

The 400°C sample shows a surprisingly very similar (and reproducible) *JV* curve to the 300°C sample. Considering the different defect landscapes between these samples (Figure 2) and the different nanorod (+ITO) resistivity values (Figure 3a), it is surprising that the *JV* curves and the *J*_sc_ and *V*_oc_ values are so similar. As far as the influence of the defects present in the ZnO nanorods on cell performance goes, the results indicate that even low temperature-annealed ZnO has sufficient quality to extract carriers away sufficiently rapidly from the cells, i.e., that *the precise defect structure within the ZnO rods is not critical to cell performance*. This finding indicates that even in far from optimally crystalline ZnO, the mobilities of the carriers in the ZnO are still far in excess of those in the blend [9,55-60].

On the other hand, the *surface defect structure and composition of the ZnO nanorods is critical to cell performance* as seen from the 200°C cell performance which is much worse than the 300°C and 400°C samples. For 200°C annealing and below, the ZnO formed at the surface of the ZnO nanorods from the decomposed Zn(OH)_2_ will be of compromised crystallinity. Also, the defects (such as zinc vacancies left behind upon OH desorption [61]) will be located near the nanorod surfaces and, hence, will give rise to surface trap states [8,62].

## Conclusions

In summary, we have presented a systematic PL study of defects present in electrodeposited ZnO as a function of annealing treatment, both in air and in Zn-rich annealing atmospheres. The relative influences of oxygen and Zn vacancies and Zn(OH)_2_ decomposition on different regions of the PL spectrum were clearly elucidated. We have confirmed that the emission wavelengths for the two main defects which make up the visible band are as follows: the oxygen vacancy emission wavelength at approximately 530 nm and the zinc vacancy emission wavelength at approximately 630 nm.

The impact of the different defect landscapes in the ZnO nanorods on the performance of ITO/ZnO/P3HT:PCBM/Ag photovoltaic devices was shown to be minimal. On the other hand, the presence of Zn(OH)_2_ on the surfaces of nanorods and its decomposition products are detrimental to photovoltaic performance. Hence, future studies on low cost, chemical solution-grown ZnO/bulk heterojunction solar cells should focus on enhancing cell performance through improving the ZnO surface quality.

## Competing interests

The authors declare that they have no competing interests.

## Authors' contributions

DCI and DM-R manufactured the nanords and solar cells. DCI collected the photoluminescence data and scanning electron microscope images. Solar cell measurements were done by DCI and DMR. BS performed the resistivity measurements of the nanorods. DCI and JLM-D planned the experiments and prepared the manuscript. QXJ assisted with photovoltaic performance analysis. All authors discussed the results and contributed to the paper drafts. All authors read and approved the final manuscript.

## References

[B1] Zhang F Xu X Tang W Zhang J Zhuo Z Wang J Wang J Xu Z Wang Y Recent development of the inverted configuration organic solar cells Sol Energ Mater Sol Cell 2011 95 1785 1799 10.1016/j.solmat.2011.02.002

[B2] Heliatek GmbHhttp://www.heliatek.com/news-19

[B3] Konarkahttp://www.konarka.com/index.php/company/our-history

[B4] Kim JY Kim SH Lee H-Y Lee K Ma W Gong X Heeger AJ New architecture for high-efficiency polymer photovoltaic cells using solution-based titanum oxide as an optical spacer Adv Mater 2006 18 572 578 10.1002/adma.200501825

[B5] Huang J-S Chou C-Y Liu M-Y Tsai K-H Lin W-H Lin C-F Solution-processed vanadium oxide as an anode interlayer for inverted polymer solar cells hybridized with ZnO nanorods Org Electron 2009 10 1060 1065 10.1016/j.orgel.2009.05.017

[B6] Gilot J Barbu I Wienk MM Janssen RAJ The use of ZnO as optical spacer in polymer solar cells: theoretical and experimental study Appl Phys Lett 2007 91 113520 10.1063/1.2784961

[B7] Yang LL Zhao QX Willander M Yang JH Ivanov I Annealing effects on optical properties of low temperature grown ZnO nanorod arrays J Appl Phys 2009 105 053503 10.1063/1.3073993

[B8] Weickert J Dunbar RB Hesse HC Wiedemann W Schmidt-Mende L Nanostructured organic and hybrid solar cells Adv Mater 2011 23 1810 1828 10.1002/adma.201003991 21509826

[B9] Olson DC Piris J Collins RT Shaheen SE Ginley DS Hybrid photovoltaic devices of polymer and ZnO nanofiber composites Thin Solid Films 2006 496 26 29 10.1016/j.tsf.2005.08.179

[B10] Takanezawa K Hirota K Wei Q-S Tajima K Hashimoto K Efficient charge collection with ZnO nanorod array in hybrid photovoltaic devices J Phys Chem C 2007 111 7218 7223

[B11] Hames Y Alpaslan Z Kösemen A San SE Yerli Y Electrochemically grown ZnO nanorods for hybrid solar cell applications Sol Energ 2010 84 426 431 10.1016/j.solener.2009.12.013

[B12] Huang J-S Chou C-Y Lee C-Y Lin C-F Synthesis and characterization of ZnO nanorod arrays and their integration into polymer solar cells IEEE Xplore: IEEE/LEOS International Conference on Optical MEMs and Nanophotonics: August 11-14 2008; Freiburg 2008 New York: IEEE 5 6

[B13] Tong H Inada M Tanaka Y Enomoto N Hojo J Preparation of nanocrystalline ZnO/TiO2 film and its application to dye-sensitized solar cells Funct Mater Lett 2012 5 2 1260006 1 10.1142/S1793604712600065

[B14] Li Q Kumar V Li Y Zhang H Marks TJ Chang RPH Fabrication of ZnO nanorods and nanotubes in aqueous solutions Chem Mater 2005 17 1001 1006 10.1021/cm048144q

[B15] Schmidt-Mende L MacManus-Driscoll JL ZnO - nanostrucutres, defects, and devices Mater Today 2007 10 40 48

[B16] McCluskey MD Jokela SJ Defects in ZnO J Appl Phys 2009 106 071101 10.1063/1.3216464

[B17] Chang C-H Huang T-K Lin Y-T Lin Y-Y Chen C-W Chu T-H Su W-F Improved charge separation and transport efficiency in poly(3-hexylthiophene)–TiO2 nanorod bulk heterojunction solar cells J Mater Chem 2008 18 2201 2207 10.1039/b800071a

[B18] Lira-Cantu M Kerbs FC Hybrid solar cells based on MEH-PPV and thin film semiconductor oxides (TiO2, Nb2O5, ZnO, CeO2 and CeO2-TiO2): performance improvement during long-time irradiation Sol Energ Mater Sol Cell 2006 90 2076 2086 10.1016/j.solmat.2006.02.007

[B19] Gershon T Metal oxide applications in organic-based photovoltaics Mater Sci Technol 2011 27 1357 1371 10.1179/026708311X13081465539809

[B20] Baxter JB Walker AM van Ommering K Aydil ES Synthesis and characterization of ZnO nanowires and their integration into dye-sensitized solar cells Nanotechnology 2006 17 S304 S312 10.1088/0957-4484/17/11/S13

[B21] Chung J Lee J Lim S Annealing effects of ZnO nanorods on dye-sensitized solar cell efficiency Physica B 2010 405 2593 2598 10.1016/j.physb.2010.03.041

[B22] Olson DC Shaheen SE Collins RT Ginley DS The effect of atmosphere and ZnO morphology on the performance of hybrid poly(3-hexylthiophene)/ZnO nanofiber photovoltaic devices J Phys Chem C 2007 111 16670 16678 10.1021/jp0734225

[B23] Pradhan D Leung KT Controlled growth of two-dimensional and one-dimensional ZnO nanostructures on indium tin oxide coated glass by direct electrodeposition Langmuir 2008 24 9707 9716 10.1021/la8008943 18652422

[B24] Guo M Yang C-Y Zhang M Zhang Y-J Ma T Wang X-D Wang X-D Effects of preparing conditions on the electrodeposition of well-aligned ZnO nanorod arrays Electrochim Acta 2008 53 4633 4641 10.1016/j.electacta.2008.01.061

[B25] Wong MH Berenov A Qi X Kappers MJ Barber ZH Illy B Lockman Z Ryan MP MacManus-Driscoll JL Electrochemical growth of ZnO nano-rods on polycrystalline Zn foil Nanotechnology 2003 14 968 973 10.1088/0957-4484/14/9/306

[B26] Pauporté T Jirka I A method for electrochemical growth of homogeneous nanocrystalline ZnO thin films at room temperature Electrochim Acta 2009 54 7558 7564 10.1016/j.electacta.2009.08.022

[B27] Khare N Kappers MJ Wei M Blamire MG MacManus-Driscoll JL Defect-induced ferromagnetism in Co-doped ZnO Adv Mater 2006 18 1449 1452 10.1002/adma.200502200

[B28] MacManus-Driscoll JL Khare N Liu Y Vickers ME Structural evidence for Zn interstitials in ferromagnetic Zn1-xCoxO films Adv Mater 2007 19 2925 2929 10.1002/adma.200602215

[B29] Heo YW Norton DP Pearton S Origin of green luminescence in ZnO thin film grown by molecular-beam epitaxy J Appl Phys 2005 98 073502 10.1063/1.2064308

[B30] Djuriić AB Ng AMC Chen XY ZnO nanostructures for optoelectronics: material properties and device applications Progr Quant Electron 2010 34 191 259 10.1016/j.pquantelec.2010.04.001

[B31] Zhou H Alves H Hofmann D Kriedseis W Meyer B Kaczmarczyk G Hoffmann A Behind the weak excitonic emission of ZnO quantum dots: ZnO/Zn(OH)2 core-shell structure Appl Phys Lett 2002 80 210 212 10.1063/1.1432763

[B32] Li Q Bian J Sun J Wang J Luo Y Sun K Yu D Controllable growth of well-aligned ZnO nanorod arrays by low-temperature wet chemical bath deposition method Appl Surf Sci 2010 256 1698 1702 10.1016/j.apsusc.2009.09.097

[B33] Bai W Zhu X Zhu Z Chu J Synthesis of zinc oxide nanosheet thin films and their improved field emission and photoluminescence properties by annealing processing Appl Surf Sci 2008 254 6483 6488 10.1016/j.apsusc.2008.04.033

[B34] Jin B Bae S Lee S Im S Effects of native defects on optical and electrical properties of ZnO prepared by pulsed laser deposition Mater Sci Eng B 2000 71 301 305 10.1016/S0921-5107(99)00395-5

[B35] Zhang X Hou S Mao H Wang J Zhu Z Influence of annealing temperature on the photoluminescence properties of ZnO quantum dots Appl Surf Sci 2010 256 3862 3865 10.1016/j.apsusc.2010.01.041

[B36] Chen Z-G Ni A Li F Cong H Cheng H-M Lu GQ Synthesis and photoluminescence of tetrapod ZnO nanostructures Chem Phys Lett 2007 434 301 305 10.1016/j.cplett.2006.12.038

[B37] Wang Q Wang G Jie J Han X Xu B Hou J Annealing effects on optical properties of ZnO films fabricated by cathodic electrodepostion Thin Solid Films 2005 492 61 65 10.1016/j.tsf.2005.06.046

[B38] Tam KH Cheung CK Leung YH Djurisic AB Ling CC Beling CD Fung S Kwok WM ChanW K Phillips DL Ding L Ge WK Defects in ZnO nanorods prepared by hydrothermal method J Phys Chem B 2006 110 20865 20871 10.1021/jp063239w 17048900

[B39] Ghotbi MY Synthesis and characterizarion of nano-sized ε-Zn(OH)2 and its decomposed product, nano-zinc oxide J Alloys Compounds 2010 491 420 422 10.1016/j.jallcom.2009.10.214

[B40] Noei H Qiu H Wang Y Löffler E Wöll C Muhler M The identification of hydroxyl groups on ZnO nanoparticles by infrared spectroscopy Phys Chem Chem Phys 2008 10 7092 7097 19039343 10.1039/b811029h

[B41] Lieth RMA Préparation and Crystal Growth of Materials with Layered Structures 1977 Boston: Kluwer

[B42] Selim FA Weber MH Solodovnikov D Lynn KG Nature of native defects in ZnO Phys Rev Lett 2007 99 085502 17930954 10.1103/PhysRevLett.99.085502

[B43] Janotti A Van de Walle CG Native point defects in ZnO Phys Rev B 2007 76 165202

[B44] Tuomisto F Saarinen K Look DC Farlow GC Introduction and recovery of point defects in electron-irradiated ZnO Phys Rev B 2005 72 085206 10.1103/physrevb.52.10932 9980192

[B45] Kuo FL Lin M-T Mensah BA Scharf TW Sheperd ND A comparative study of the photoluminescence and conduction mechanisms of low temperature pulsed laser deposited and atomic layer deposited zinc oxide thin films Phys Status Solidi A 2010 11 1 5

[B46] Liao Z-M Zhang H-Z Zhao Y-B Xu J Zhang J-M Yu DP Surface effects on photoluminescence of single ZnO nanowires Phys Lett A 2008 372 4505 4509 10.1016/j.physleta.2008.04.013

[B47] Xu PS Sun YM Shi CS Xu FQ Pan HB The electronic structure and spectral properties of ZnO and its defects Nucl Instrum Meth Phys Res B 2003 199 286 290

[B48] Lin B Fu Z Jia Y Green luminescent center in undoped zinc oxide films deposited on silicon substrates Appl Phys Lett 2001 79 943 945 10.1063/1.1394173

[B49] Kim Y-S Park CH Rich variety of defects in ZnO via an attractive interaction between O vacancies and Zn interstitials: origin of n-type doping Phys Rev Lett 2009 102 086403 19257760 10.1103/PhysRevLett.102.086403

[B50] Seo H Park C-J Cho Y-J Kim Y-B Choi D-K Correlation of band edge native defect state evolution to bulk mobility changes in ZnO thin films Appl Phys Lett 2010 96 232101 10.1063/1.3424790

[B51] Trejo-Cruz C Mendoza-Galvan A Lopez-Beltran AM Garcia-Jimenez M Effects of air annealing on the optical, electrical, and structural properties of indium-tin oxide thin films Thin Solid Films 2009 517 4615 4620 10.1016/j.tsf.2009.02.134

[B52] Kawashima T Ezure T Okada K Matsui H Goto K Tanabe N FTO/ITO double-layered transparent conductive oxide for dye-sensitized solar cells J Photochem Photobiol Chem 2004 164 199 202 10.1016/j.jphotochem.2003.12.028

[B53] Gregory OJ Amani M Tougas IM Drehman AJ Stability and microstructure of indium tin oxynitride thin films J Am Ceram Soc 2012 95 705 710 10.1111/j.1551-2916.2011.04845.x

[B54] Kim Y-N Shin H-G Song J-K Cho D-H Lee H-S Jung Y-G Thermal degradation behavior of indium tin oxide thin films deposited by radio frequency magnetron sputtering J Mater Res 2005 20 1574 1579 10.1557/JMR.2005.0199

[B55] Cui J Zinc oxide nanowires Mater Charact 2012 64 43 52

[B56] Fan Z Wang D Chang P-C Tseng W-Y Lu JG ZnO nanowire field-effect transistor and oxygen sensing property Appl Phys Lett 2004 85 5923 5925 10.1063/1.1836870

[B57] Rakhshani AE Optical and electrical characterization of well-aligned ZnO rods electrodeposited on stainless steel foil Appl Phys A 2008 92 303 308 10.1007/s00339-008-4526-y

[B58] Ma H Yip H-L Huang F Jen AK-Y Interface engineering for organic electronics Adv Func Mater 2010 20 1371 1388 10.1002/adfm.200902236

[B59] Vanlaeke P Sinnen A Haeldemans I Vanhoyland G Aernouts T Cheys D Deibel C D’Haen J Heremans P Poortmans J Manca JV P3HT/PCBM bulk heterojunction solar cells: relation between morphology and electro-optical characteristics Sol Energ Mat Sol Cell 2006 90 2150 2158 10.1016/j.solmat.2006.02.010

[B60] MacKenzie RCI Kirchartz T Dibb GFA Nelson J Modeling nongeminate recombination in P3HT:PCBM solar cells J Phys Chem C 2011 115 9806 9813 10.1021/jp200234m

[B61] Bera A Basak D Role of defects in the anomalous photoconductivity in ZnO nanowires Appl Phys Lett 2009 94 163119 10.1063/1.3123167

[B62] Snaith HJ Schmidt-Mende L Advances in liquid-electrolyte and solid-state dye-sensitized solar cells Adv Mater 2006 19 3187 3200

